# *In situ* reduction of gold nanoparticles-decorated MXenes-based electrochemical sensing platform for KRAS gene detection

**DOI:** 10.3389/fbioe.2023.1176046

**Published:** 2023-03-17

**Authors:** Xiongtao Yu, Silan Bai, Lishi Wang

**Affiliations:** School of Chemistry and Chemical Engineering, South China University of Technology, Guangzhou, China

**Keywords:** MXenes, gold nanoparticle, biomarker, CtDNA, electrochemical, biosensor

## Abstract

In this work, gold nanoparticles@Ti_3_C_2_ MXenes nanocomposites with excellent properties were combined with toehold-mediated DNA strand displacement reaction to construct an electrochemical circulating tumor DNA biosensor. The gold nanoparticles were synthesized *in situ* on the surface of Ti_3_C_2_ MXenes as a reducing and stabilizing agent. The good electrical conductivity of the gold nanoparticles@Ti_3_C_2_ MXenes composite and the nucleic acid amplification strategy of enzyme-free toehold-mediated DNA strand displacement reaction can be used to efficiently and specifically detect the non-small cell cancer biomarker circulating tumor DNA KRAS gene. The biosensor has a linear detection range of 10 fM −10 nM and a detection limit of 0.38 fM, and also efficiently distinguishes single base mismatched DNA sequences. The biosensor has been successfully used for the sensitive detection of KRAS gene G12D, which has excellent potential for clinical analysis and provides a new idea for the preparation of novel MXenes-based two-dimensional composites and their application in electrochemical DNA biosensors.

## Introduction

In recent years, Ti_3_C_2_ MXenes, a new type of two-dimensional layered nanomaterial has received much attention because of its excellent properties such as good electrical conductivity (up to 2.4 × 10^5^ S/m), large surface area, easy film formation, and good biocompatibility (Yuan et al., 2018; Zhang et al., 2018; Pang et al., 2019). Ti_3_C_2_ MXenes have a wide range of promising applications in catalysis (Li and Wu, 2019; Su et al., 2019), environmental protection (Lim et al., 2020), biosensors (Wen et al., 2017; Kumar et al., 2018; Wang et al., 2020; Lu et al., 2021), supercapacitors (Wang et al., 2018; Chen et al., 2021), batteries (Li et al., 2021; Lee et al., 2022), etc. Based on these excellent properties, Ti_3_C_2_ MXenes have great potential for sensor construction.

MXenes compounded with other nanomaterials provide better performance and enhance the detection performance of the sensing platform (Song et al., 2022; Yang et al., 2022). Ti_3_C_2_ MXenes-based composites could be used in sensing platforms not only as carriers of signal probes but also to facilitate interfacial electron transfer (Chen et al., 2013; Wang et al., 2013). Compounding of Ti_3_C_2_ MXenes with metal nanoparticles or metal oxides is more favored in the synthesis of Ti_3_C_2_ MXenes-based composites. Metal nanoparticles, metal oxides, and other nanomaterials are synthesized by adding stabilizers such as surfactants to avoid aggregation. However, surfactants may cover the surface of the nanomaterials, thus obscuring the active sites and blocking electron transfer, which may adversely affect the performance of the sensors. (Steigerwalt et al., 2002; Bing et al., 2010; Dey et al., 2013). Therefore, nanomaterials without surfactants with excellent conductivity may be more suitable for the construction of electrochemical sensors. Ti_3_C_2_ MXenes have great potential for the preparation of MXenes-metal nanoparticle composites due to their strong reducing ability. For example, the synthesized composites MXenes/magnetic iron oxide and MXenes/Ag have a strong catalytic capacity (Zhang et al., 2016; Zou et al., 2016). Ti_3_C_2_ MXenes are widely used in the synthesis of surfactant-free nanomaterials due to their unique properties and simple preparation process.

Liquid biopsy is a method of sampling and analyzing body fluids such as blood, urine, and saliva to detect and diagnose cancer or other diseases. Liquid biopsy can detect a range of biomarkers such as circulating tumor DNA (ctDNA), circulating tumor cells, and exosomes (Wu et al., 2020). CtDNA is a biomarker for tumor cells to release their DNA fragments in body fluids. Breakthroughs in ctDNA analysis and detection technologies are driving the development of minimally invasive liquid biopsy for disease (Das et al., 2016). Therefore, ctDNA detection has received a lot of attention in the field of tumor diagnosis and treatment. Colorectal cancer (CRC) is a type of gastrointestinal malignancy with a high mortality rate and an increasing incidence, currently causing at least 890,000 deaths per year (Bray et al., 2018; Siegel et al., 2020; Wang et al., 2022). Mutation and activation of the KRAS gene in the human body are important causes of the development and progression of colorectal cancer (Shaukat et al., 2012). The KRAS gene is often mutated at codons 12 and 13 and is an unfavorable factor in the development, progression, and prognosis of colorectal cancer (Zhu et al., 2007). Therefore, the detection and evaluation of the KRAS gene is an important tool for the early diagnosis and treatment of colorectal cancer. Commonly used analytical methods in clinical settings include polymerase chain reaction (PCR), mass spectrometry, surface-enhanced Raman scattering (SERS), and next-generation sequencing (NGS). Although these methods have some advantages, the development of low-cost, simple, sensitive, and portable nucleic acid detection methods remains a challenge. Electrochemical methods have been widely used in clinical diagnostics, environmental monitoring, food safety, and immunoassays due to their high sensitivity, short time consumption, ease of control, and low cost. (Yang et al., 2016; Zheng et al., 2020; Yu et al., 2022). On the other hand, many analytical techniques including some electrochemical methods require enzyme participation to improve sensitivity. However, the non-specificity of enzyme-catalyzed reaction and the need for harsh reaction conditions bring difficulties to the detection technology (Yang F. et al., 2021; Yang J. et al., 2021; Zhuang et al., 2021). The toehold-mediated DNA strand displacement reaction (TMSD) is widely used in the construction of biosensors due to its ability to amplify signals without the involvement of enzymes and its high reaction efficiency and simple design (Zhang et al., 2020a; Zhang et al., 2020b; Bialy et al., 2021).

In this study, a large number of AuNPs were synthesized *in situ* on the surface of Ti_3_C_2_ MXenes as a reducing and stabilizing agent, thus successfully preparing two-dimensional nanocomposite AuNPs@Ti_3_C_2_ MXenes with excellent electrochemical properties. The large surface area of Ti_3_C_2_ MXenes can be loaded with a lot of AuNPs and then self-assembled with more DNA double-strand probes *via* Au-S bonds thus efficiently facilitating the chain substitution reaction. The KARS G12D electrochemical DNA biosensor was constructed using the excellent electrochemical properties of AuNPs@Ti_3_C_2_ MXenes combined with nucleic acid amplification strategy of enzyme-free toehold-mediated DNA strand displacement reaction. The designed sensor exhibits excellent sensitivity and can be applied to the analysis of serum samples.

## Materials and methods

### Materials

Ti_3_AlC_2_ MAX was acquired from Aladdin (Shanghai, China). Tris (hydroxymethyl)aminomethane (Tris), and 6-Mercapto-1-hexanol (MCH) were acquired from Sigma. Tris (2-carboxyethyl) phosphine hydrochloride (TCEP) and chloroauric acid (HAuCl_4_·4H_2_O, ≥99.9%) were ordered from J&K scientific. Hydrofluoric acid was acquired from Sinopharm Chemical Reagent Co., Ltd. (Shanghai, China). 0.1 M pH 7.4 Tris-HCl buffer (100 mM NaCl and 20 mM MgCl_2_) was used for electrochemical measurement, and 10 mM pH 7.4 Tris-HCl buffer was used as washing buffer. All DNA strands were ordered from Sangon Biotech Co., Ltd. (Shanghai, China), and the DNA sequences were provided in Table 1. 18.2 MΩ cm ultrapure water was used in whole experiments.

**TABLE 1:** A list of the oligonucleotide sequences.

DNA	Sequence (5′-3′)
template DNA	SH-(CH_2_)_6_^—^GAAATGGTGGAAAGGTCAACTG
	GAGCTGGTGGCGTAG
assisted DNA	CAC​CAG​CTC​CAG​TTG​ACC​CTA​TAT​CCA​TAA
protected DNA	CCTTTCCACCATTTC
probe DNA	CAC​CAG​CTC​CAG​TTG​ACC​TTT​CCA​CCA​TTT​C- methylene blue
KARS G12D	CTACGCCACCAGCTCCA
Single-base mismatch (1 M)	CTA*T*GCCACCAGCTCCA
Two-base mismatch (2 M)	CTA*T*GC*A*ACCAGCTCCA
Three-base mismatch (3 M)	CTA*T*GC*A*AC*G*AGCTCCA
Random DNA	AGCATTGACTACGCCGT

### Apparatus

Scanning electron microscopy (SEM) images and elemental mapping were characterized by SU8220 field-emission scanning electron microscope (Hitachi Ltd, Japan, 10.0 kV). X-ray diffraction (XRD) was characterized by X’pert Powder X-ray diffraction (Panalytical. Ltd, Netherlands). Transmission electron microscope (TEM) images were recorded on a JEM-2100F field emission electron microscope (JEOL, Japan) at an accelerating voltage of 200 kV.

### Preparation of Ti_3_C_2_ MXenes

**SCHEME 1:**
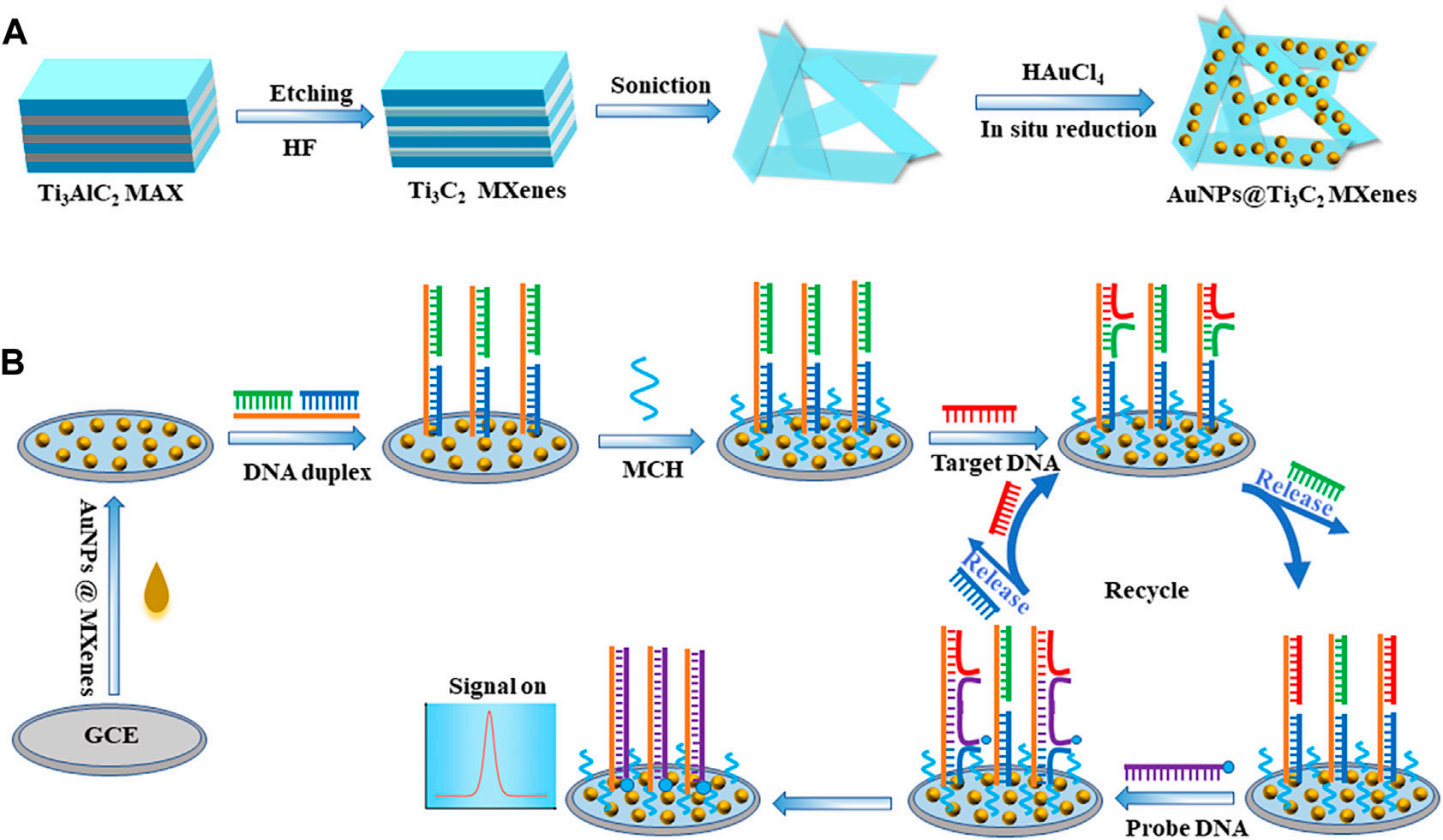
Schematic illustrations of **(A)** the synthesis route for AuNPs@Ti3C2 MXenes nanocomposites, and **(B)** electrochemical sensing platform for KRAS gene detection via toehold-mediated strand displacement reaction.

Ti_3_C_2_ MXenes were prepared by etching the Al element in Ti_3_AlC_2_ MAX with HF solution referring to the previous literature with minor modifications (Lin et al., 2020) (Scheme 1A). Briefly, 1 g Ti_3_AlC_2_ was added to 50 mL 45% HF solution, and then etched at room temperature for 24 h. The precipitate was collected by centrifugation at 6,000 rpm for 15 min. Finally, the precipitate was washed several times with deionized water until the pH of the supernatant exceeded 6. The Ti_3_C_2_ MXenes were obtained.

### Synthesis of AuNPs@Ti_3_C_2_ MXenes nanocomposites

AuNPs@Ti_3_C_2_ MXenes nanocomposites were synthesized referring to the previous literature with minor modifications (Mi et al., 2021; Song et al., 2022) (Scheme 1A). AuNPs were synthesized by *in situ* reduction on the surface of Ti_3_C_2_ MXenes and Ti_3_C_2_ MXenes were used as the reducing agent and support material. Firstly, 15 mg pre-prepared Ti_3_C_2_ MXenes were well-dispersed in 45 mL deionized water, and the Ti_3_C_2_ suspension (0.33 mg mL^−1^) was prepared after sonicating for 4 h. Then, 600 μL of 1 g mL^−1^ HAuCl_4_ solution was slowly added to the Ti_3_C_2_ suspension drop by drop with gentle stirring. After the reaction at room temperature for 30 min, the resulting suspension was centrifuged at 8,000 rpm for 10 min and the precipitate was collected. The AuNPs@Ti_3_C_2_ two-dimensional nanocomposites were successfully prepared by washing with deionized water several times for subsequent studies.

### Fabrication of DNA biosensor

The bare glassy carbon electrode (GCE) was polished with 0.05 μm alumina slurry for 5 min before modification and then sonicated in ethanol and deionized water for 2 min, respectively. Finally, the bare GCE was rinsed with deionized water and then dried with nitrogen gas for the following experiment. Subsequently, 1 mg mL^−1^ AuNPs@Ti_3_C_2_ MXenes nanocomposites were dropped onto the GCE surface to obtain the AuNPs@Ti_3_C_2_ MXenes/GCE and dried naturally. 5 μM template DNA, 5 μM protected DNA and 5 μM auxiliary DNA were incubated at 90°C for 5 min, and then gradually cooled to room temperature to form stable DNA duplex structures. Then, a final concentration of 10 mM of TCEP was added to the DNA duplex for 1 hour at room temperature to break the disulfide bond. Subsequently, drop 10 μL 1 μM DNA double-strand probes onto the modified electrode AuNPs@Ti_3_C_2_ MXenes/GCE and incubate at room temperature for 3 h to form self-assembled monolayers. The electrodes were immersed in 1 mM MCH to block the unbound sites, then washed with Tris-HCl buffer and dried with nitrogen subsequently.

### Detection of KARS G12D

The electrodes of the modified DNA duplex probes were washed with buffer and then immersed in a solution containing 50 μL^1^ μM probe DNA and various concentrations of target DNA KARS G12D. The TMSD recycling process took 2 hours to complete at room temperature. Subsequently, the electrodes were washed with 10 mM Tris-HCl buffer and were used for electrochemical measurement.

### Electrochemical measurements

A CHI660E electrochemical workstation (Shanghai Chenhua Instrument Co. Ltd., China) was used for all electrochemical measurements which contained a three-electrode system consisting of a modified 3 mm GCE, a platinum auxiliary electrode, and a saturated Ag/AgCl reference electrode. Square wave voltammetry (SWV) experiments were carried out in 10 mM Tris–HCl buffer (100 mM NaCl and 20 mM MgCl_2_, pH 7.4) using the following parameters: a potential range of −0.6 to 0 V, an amplitude of 25 mV, a frequency of 25 Hz, and a step potential of 4 mV. To prevent the interference of oxygen reduction during the electrochemical measurement, high-grade nitrogen should be used to purge the detection buffer for 30 min. Electrochemical impedance spectroscopy (EIS) was performed in 5 mM [Fe (CN)_6_]^3−/4−^ contained 0.1 M KCl, in frequency range from 0.03 Hz to 10 kHz with the bias potential of 0.192 V and the amplitude of 5 mV, respectively.

## Results and discussion

### Characterizations of the Ti_3_C_2_ MXenes and AuNPs@Ti_3_C_2_ MXenes nanocomposites

**FIGURE 1:**
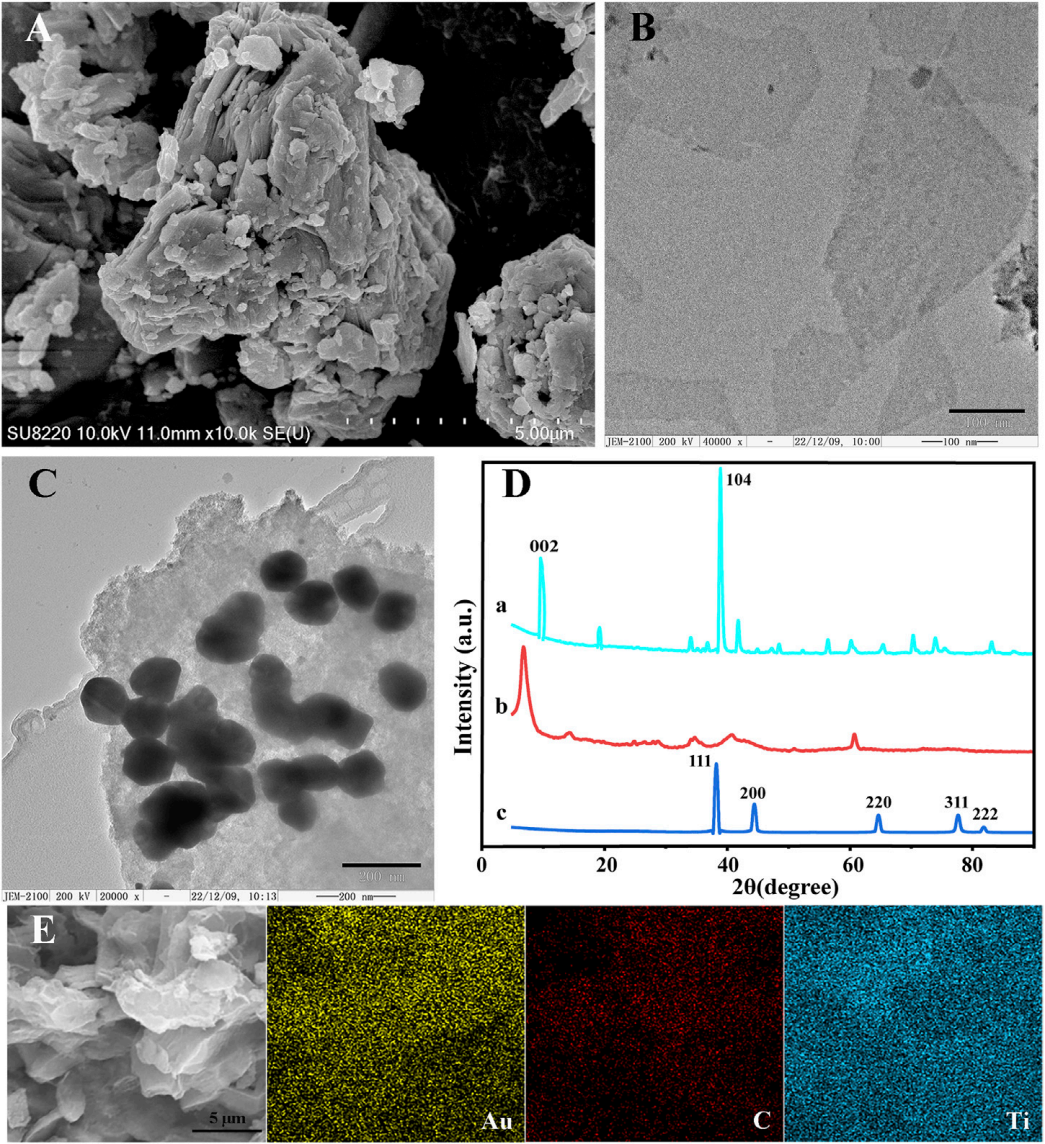
SEM images of Ti_3_AlC_2_ **(A)**. TEM images of Ti_3_C_2_ **(B)** and AuNPs@Ti_3_C_2_ MXenes **(C)**. **(D)** XRD of Ti_3_AlC_2_ **(a)**, Ti_3_C_2_ **(b)**, AuNPs@Ti_3_C_2_ **(c)**. **(E)** Elemental mapping images of AuNPs@Ti_3_C_2_.

The morphologies of Ti_3_AlC_2,_ Ti_3_C_2,_ and AuNPs@Ti_3_C_2_ MXenes were characterized by SEM and TEM. Ti_3_AlC_2_ without etching treatment exhibited an irregular morphology (Figure 1A). The Ti_3_C_2_ MXenes prepared by etching and ultrasound show a smooth, thin, and flake structure (Figure 1B) due to the disappearance of the Al layer after the HF etching. Figure 1C shows that the size of AuNPs synthesized by *in situ* reduction on the surface of Ti_3_C_2_ is about 125 nm. Because of the large surface area of Ti_3_C_2_, a lot of AuNPs were synthesized. By measuring the elemental properties of AuNPs@Ti_3_C_2_ MXenes, the results showed that the Ti, C, and Au elements of AuNPs@Ti_3_C_2_ MXenes were uniformly distributed and continuously (Figure 1E).

The XRD characterization further determined the composition and crystal structure of the nanocomposites (Figure 1D). The diffraction peaks of Ti_3_AlC_2_ at 9.5°, 19.06°, 38.7°, and 41.84° matched well with the diffraction peaks of the standard card (JCPDS card number: 52-0875) (curve a in Figure 1D). The XRD results of the Ti_3_C_2_ showed the disappearance of the 38.7° diffraction peak in the Al (104) plane and the left shift of the diffraction peak in the (002) plane of Ti_3_C_2_ from 9.5° to 8.27°, which indicated the successful preparation of Ti_3_C_2_ (curve b in Figure 1D) (Ran et al., 2017; Alhabeb et al., 2018; Meng et al., 2021). When AuNPs are generated by *in situ* reduction on the surface of Ti_3_C_2_, curve c in Figure 1D shows that AuNPs@Ti_3_C_2_ have the characteristic diffraction peaks of both Ti_3_C_2_ and AuNPs (Yang et al., 2022). The SEM images, TEM images, mapping, and XRD results indicated that the two-dimensional nanocomposite AuNPs@Ti_3_C_2_ MXenes were successfully synthesized.

### The construction of electrochemical biosensor based on TMSD and AuNPs@Ti_3_C_2_ MXenes nanocomposites

Toehold-mediated strand displacement reaction was used in the development of many biosensors due to its high specificity and without the involvement of enzymes. In the toehold-mediated strand displacement reaction, one oligonucleotide hybridizes with the toehold domain of double-strand DNA resulting in the dissociation of the substrate strand from the double-strand DNA (Irmisch et al., 2020; Li et al., 2023). In this study, we developed two efficient and simple toehold-mediated strand displacement reactions (Scheme 1B). The first toehold-mediated strand displacement reaction was performed when target DNA KARS G12D hybridizes with the double-strand DNA probes. The second toehold-mediated strand displacement reaction was performed when the signal probes were present in the reaction system, and the dissociated target DNA KARS G12D entered the next cycle of reaction. The fabrication of the DNA biosensor based on toehold-mediated strand displacement reaction was illustrated in Scheme 1. First, the pre-prepared AuNPs@Ti_3_C_2_ MXenes were modified on the GCE. Then double-strand DNA probes were self-assembled onto the AuNPs surface *via* Au-S bonding. The toehold of strand displacement reaction was formed. Subsequently, the target DNA KARS G12D and the signal probe were added for strand displacement reactions to achieve circular hybridization of KARS G12D. Meanwhile, the signal probe was linked to the electrode by the second strand displacement reaction to generate an electrochemical signal.

**FIGURE 2:**
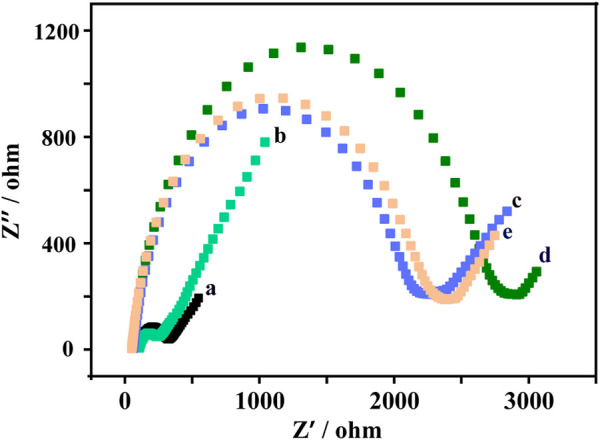
Electrochemical impedance spectroscopy (EIS) response of bare GCE **(a)**, gold nanoparticles @ Ti_3_C_2_ MXenes/GCE **(b)**, DNA double-strand probes/gold nanoparticles @Ti_3_C_2_ MXenes/GCE **(c)**, MCH/DNA double-strand probes/gold nanoparticles@ Ti_3_C_2_ MXenes/GCE **(d)**, probe DNA/target DNA/MCH/DNA double-strand probes/gold nanoparticles@Ti_3_C_2_ MXenes/GCE **(e)** in 5 mM [Fe(CN)_6_]^3−/4−^ containing 0.1 M KCl.

The EIS measurement results were shown in Figure 2. The bare glassy carbon electrode shows a small Ret value (curve a). When the bare glassy carbon electrode was modified with AuNPs@Ti_3_C_2_ MXenes composites, the Ret value became smaller (curve b), indicating that AuNPs@Ti_3_C_2_ MXenes composites have excellent electrical conductivity. The Ret value gradually increased when the DNA duplex structure and MCH were gradually modified, which was caused by the low conductivity of the DNA duplex structure and MCH (curves c and d). When the TMSD occurred after adding probe DNA and KARS G12D, the Ret value became smaller, probably due to the reduction of a steric hindrance after the hybridization of probe DNA and template DNA (curve e). The results of EIS and PAGE (Supplementary Figure S1) demonstrate the successful modification of AuNPs@Ti_3_C_2_ MXenes composites on glassy carbon electrodes and the successful design of TMSD.

### Analytical performance of AuNPs@Ti_3_C_2_ MXenes-based biosensor

**FIGURE 3:**
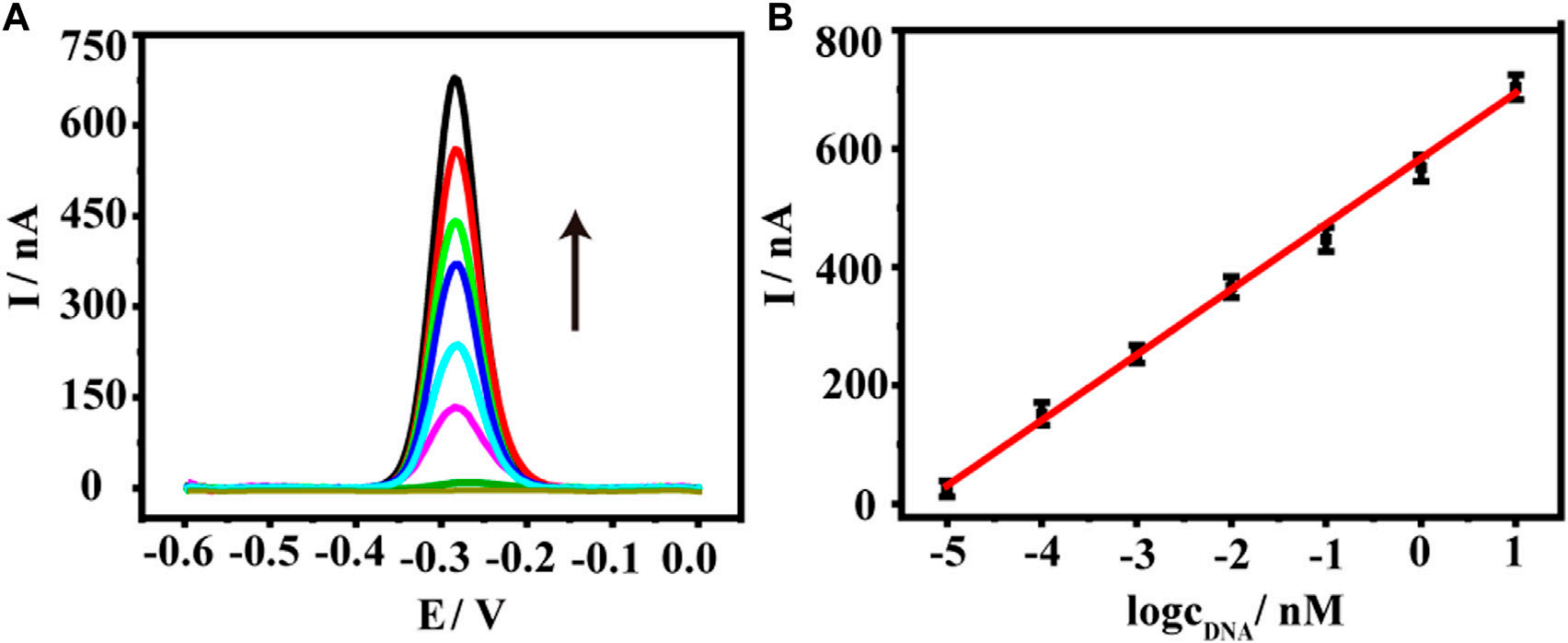
**(A)** Square wave voltammetry (SWV) curves of the DNA biosensor to KARS G12D gene at a series of concentrations, from down to top (black arrow): 0, 10^−5^, 10^−4^,10^−3^, 10^−2^, 10^−1^, 1, 10 nM. **(B)** The logarithmic plot of the current value of the oxidation peak versus the KARS G12D gene concentrations from 10^−5^ nM–10 nM. Error bars represent the standard deviations of three independent experiments.

We thus used the sensor to detect a range of KARS G12D at concentrations from 10^−5^ nM–10 nM. As shown in Figure 3A, as the concentration of KARS G12D increased, the electrochemical signal values of methylene blue progressively increased, showing a typical concentration-dependent event. Further quantitative analysis exhibited a logarithmic relationship between the electrochemical signal values of methylene blue and the concentration of KARS G12D (Figure 3B). The calibrated regression equation was fitted to y (nA) = 110.6 log C_tDNA_ + 583.4 (R^2^ = 0.9946), with a detection limit of 0.38 fM (S/N = 3). Such a low detection limit was equivalent to many enzyme-free sensors or even those sensors using enzymes. The constructed DNA sensor has high sensitivity and a wide linear range (Supplementary Table S1).

### Specificity, stability, and reproducibility of DNA sensor

**FIGURE 4:**
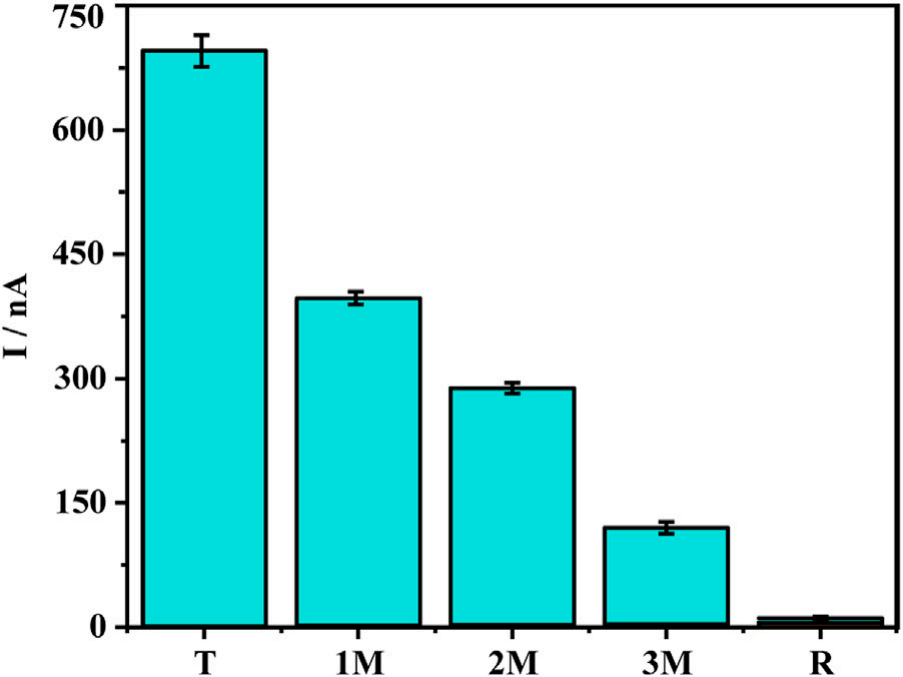
Specificity investigation of five different DNA sequences: Target DNA KARS G12D (T), single-base mismatched DNA (1 M), two-base mismatched DNA (2 M), three-base mismatched DNA (3 M), random DNA sequence (R). Error bars represent the standard deviations of three independent experiments.

Since the sensor has good detection performance, we further test the specificity of the sensor. Point mutations increase the difficulty of gene detection because they cause changes in the characteristics of genes. Therefore, the ability to identify point mutations is an important parameter for gene detection technology (Chang et al., 2015; Yu et al., 2022). We verified the specificity of the DNA sensor using five types of DNA sequences, including target DNA KARS G12D (T), single-base mismatched DNA (1 M), double-base mismatched DNA (2 M), triple-base mismatched DNA (3 M) and random sequence DNA (R) (Figure 4). A comparison of the oxidation peak current values of methylene blue revealed the current value of the 1 M group was much smaller than the T group, only 57% of the T group, revealing the ability of the sensor to recognize single base mismatches effectively. The oxidation peak current values of 2 M and 3 M were only 41% and 17% of the target DNA, while the peak current values of the random sequence DNA were almost close to the background values. To further ensure the performance of the sensor, the reproducibility and stability were also tested. The relative standard deviation (RSD) of the current signal values for the four parallel electrodes was 3.59%. In addition to this, the current signal value of the treated electrodes after 7 days at 4°C was 96.4% of the original value. The above results indicate that the sensor has good specificity, reproducibility, stability, and has good potential for clinical applications.

### Real sample analysis

The sensor has good sensitivity, specificity, and stability. We further evaluated its ability to be used in clinical practice with real samples. Different concentrations of KARS G12D were added to a 10-fold dilution of healthy human serum for recovery testing. The recoveries of different concentrations of KARS G12D were 109%, 100.1%, and 93.3%, respectively (Table 2). The results showed that the sensor has good potential for monitoring ctDNAs in complicated biological samples.

**TABLE 2:** Detection of KARS G12D gene added in serum samples.

Sample	Added (pM)	Found (pM)	Recovery (%)
1	1	1.09	109
2	5	5.03	100.1
3	10	9.33	93.3

## Conclusion

In this work, we constructed an electrochemical DNA biosensor based on two-dimensional nanocomposite AuNPs@Ti_3_C_2_ MXenes and a non-enzymatic toehold-mediated strand displacement reaction. The good electrical conductivity of AuNPs@Ti_3_C_2_ MXenes and the signal amplification strategy of toehold-mediated strand displacement reaction can be used to detect KRAS G12D with high efficiency and specificity. The biosensor has excellent detection performance with a detection limit of 0.38 fM, and also effectively distinguishes single-base mismatched DNA sequences. In addition to detecting KRAS G12D, the platform also allows for rapid and easy detection of other disease biomarkers by redesigning disease-related DNA probes. The novel 2D composite material based on MXenes has good potential for application in biosensing platforms.

## Data Availability

The original contributions presented in the study are included in the article/Supplementary Material, further inquiries can be directed to the corresponding authors.
