# Supplementary material for: In situ reduction of gold nanoparticles-decorated MXenes-based electrochemical sensing platform for KRAS gene detection
Source: Front Bioeng Biotechnol. 2023 Mar 17;11:1176046. doi: 10.3389/fbioe.2023.1176046 (PMC10063977; doi:10.3389/fbioe.2023.1176046)
Supplement: Supplementary file 1 [file DataSheet1.docx]

Supplementary Material

The migration rate of DNA in PAGE slows down with increasing molecular weight. As shown in Figure S1, lane 1 is 20 bp DNA Ladder. Template DNA, KARS G12D, assisted DNA, probe DNA and protected DNA show a single band of DNA (lanes 2,3,4,5,6) respectively. After the mixing of template DNA, assisted DNA and protected DNA, a single band with slow migration was observed in lane 7, indicating the successful formation of double-stranded DNA probe. When KARS G12D was added to the solution in which the double-stranded DNA probe had been formed, a band with the brightest color and smaller molecular weight than the band in lane 7 was observed (lane 8), indicating that the assisted DNA was released after hybridization of the KARS G12D with the double-stranded DNA probe. Finally, when probe DNA was added to the solution, KARS G12D, assisted DNA, and protected DNA were all released (lane 9). The PAGE results demonstrated that the toehold-mediated strand displacement reaction occurred successfully.


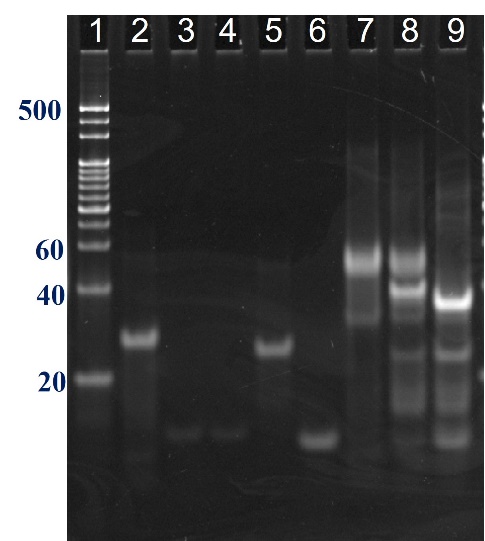


Figure S1 The image of polyacrylamide gel electrophoresis of toehold-mediated strand displacement reaction signal amplification.

lane 1: 20bpDNA Ladder, lane 2: template DNA, lane 3: target DNA, lane 4: assisted DNA, lane 5: probe DNA, lane 6: protected DNA, lane 7: template DNA + assisted DNA + protected DNA, lane 8: template DNA + assisted DNA + protected DNA + target DNA, lane 9: template DNA + assisted DNA + protected DNA + target DNA + probe DNA. Gel Red nucleic acid stain was used in the experiment.

Table S1. Comparison of sensor performance in detecting KRAS G12D with reported literature

| Method | Amplification strategy | Linear range | LOD | Reference |
| --- | --- | --- | --- | --- |
| Flu ^a^ | dPCR | 5~50 ng/mL | 0.05% | (Pratt et al., 2019) |
| SERS | THMS | 10 fM~1 nM | 0.3 fM | (Zhou et al., 2016) |
| PEC | Enzyme-free | 0.5 fM~100 fM | 0.2 fM | (Yang et al., 2022) |
| EC | alDNA | 0.1 pM~10 nM | _ | (Zeng and Xiang, 2019) |
| EC | TMSD | 10 fM -10 nM | 0.38 fM | This work |

^a^ Flu: fluorescence, ddPCR: digital droplet polymerase chain reaction, SERS: surface-enhanced Raman scattering, THMS: the triple-helix molecular switch structure, PEC: photoelectrochemical, EC: electrochemical, alDNA: anchor-like DNA, TMSD: toehold-mediated DNA strand displacement

^b^ 0.05%: abundance

Pratt, E.D., Cowan, R.W., Manning, S.L., Qiao, E., Cameron, H., Schradle, K., et al. (2019). Multiplex Enrichment and Detection of Rare KRAS Mutations in Liquid Biopsy Samples using Digital Droplet Pre-Amplification. *Analytical Chemistry* 91(12)**,** 7516-7523. doi: 10.1021/acs.analchem.8b01605.

Yang, X., Zhao, L., Lu, L., Feng, M., Xia, J., Zhang, F., et al. (2022). In Situ Reduction of Gold Nanoparticle-Decorated Ti3C2 MXene for Ultrasensitive Electrochemical Detection of MicroRNA-21 with a Cascaded Signal Amplification Strategy. *Journal of The Electrochemical Society* 169(5). doi: 10.1149/1945-7111/ac6a7f.

Zeng, N., and Xiang, J. (2019). Detection of KRAS G12D point mutation level by anchor-like DNA electrochemical biosensor. *Talanta* 198**,** 111-117. doi: <https://doi.org/10.1016/j.talanta.2019.01.105>.

Zhou, Q., Zheng, J., Qing, Z., Zheng, M., Yang, J., Yang, S., et al. (2016). Detection of Circulating Tumor DNA in Human Blood via DNA-Mediated Surface-Enhanced Raman Spectroscopy of Single-Walled Carbon Nanotubes. *Analytical Chemistry* 88(9)**,** 4759-4765. doi: 10.1021/acs.analchem.6b00108.
